# Telerehabilitation in Postoperative Breast Cancer Care: Systematic Review and Meta-Analysis

**DOI:** 10.2196/77161

**Published:** 2025-12-09

**Authors:** Fabio Santacaterina, Benedetta Campagnola, Federica Bressi, Loredana Zollo, Silvia Sterzi, Marco Bravi

**Affiliations:** 1 Rehabilitation Fondazione Policlinico Universitario Campus Bio-Medico Rome Italy; 2 Department of Engineering, Research Unit of Advanced Robotics and Human-Centred Technologies Università Campus Bio-Medico Rome Italy; 3 Department of Medicine and Surgery Università Campus Bio-Medico Rome Italy; 4 Fondazione Policlinico Universitario Campus Bio-Medico Rome Italy

**Keywords:** telerehabilitation, physiotherapy, breast cancer, postoperative care, quality of life, pain, upper limb function, randomized controlled trial

## Abstract

**Background:**

Postoperative rehabilitation is essential to improve quality of life (QoL), pain control, and upper limb function in women undergoing surgery for breast cancer (BC). Telerehabilitation has emerged as a promising alternative to conventional rehabilitation, especially in patients with limited access to care, but its comparative efficacy remains uncertain.

**Objective:**

This study aimed to evaluate the effectiveness of telerehabilitation compared with standard care or no treatment in improving QoL, pain, handgrip strength, and upper limb function in women undergoing BC surgery.

**Methods:**

We conducted a systematic review and meta-analysis following PRISMA (Preferred Reporting Items for Systematic Reviews and Meta-Analyses) 2020 guidelines. We included 11 randomized controlled trials, of which 5 were eligible for quantitative synthesis. Risk of bias was assessed using the Cochrane risk-of-bias tool for randomized trials version 2 (RoB2), and the certainty of evidence was evaluated using the Grading of Recommendations Assessment, Development and Evaluation approach. Outcomes assessed included QoL, pain, grip strength, and upper limb function.

**Results:**

Telerehabilitation significantly improved QoL (standardized mean difference [SMD] 0.59; 95% CI 0.24-0.95; moderate certainty) and grip strength (mean difference [MD] 2.93; 95% CI 0.82-5.04 kg; low certainty), and significantly reduced pain (SMD –0.50; 95% CI –0.79 to –0.22; low certainty). No significant difference was observed for upper limb function (SMD –0.86; 95% CI –2.02 to 0.31; low certainty).

**Conclusions:**

Telerehabilitation is an effective and viable intervention for improving QoL, reducing pain, and enhancing grip strength in women following BC surgery. However, its impact on upper limb function remains inconclusive and requires further investigation.

**Trial Registration:**

PROSPERO CRD42024545075; https://www.crd.york.ac.uk/PROSPERO/view/CRD42024545075

## Introduction

Breast cancer (BC) is the most frequently diagnosed cancer in women: an incidence analysis of 2020 reported that BC became the most frequently diagnosed cancer worldwide, with an estimation of 2.26 million of new cases [1]. In Italy, it is the neoplasm with the highest incidence and represents the main cause of death from cancer in women [2]. Therefore, some recent predictions and analyses [3,4] suggested that the incidence of new cases will increase to over 3 million cases annually by 2040. Due to its high incidence and future predictions, BC is considered a cause for global concern.

Advances in early diagnosis and treatment have led to increased survival after diagnosis, resulting in many more women living with the outcomes of surgical treatment of the condition. The disorders most frequently encountered after breast surgery are postoperative pain (68% of patients), followed by musculoskeletal problems and functional limitations in the shoulder (59% of the population after mastectomy and quadrantectomy) and a reduction in range of motion (ROM) and muscle strength in 24%-53% [5]. Other complications reported include “axillary web syndrome,” which can cause pain and movement limitations [6]; lymphedema reported with a prevalence of 6%-52% especially after axillary lymph node dissection; kinematic alterations that may contribute to the future development of rotator cuff tendinopathy [7]; scapulohumeral dyskinesia, proprioception deficit, postural alterations and reduction in perceived quality of life (QoL) [8].

Literature suggests that early postoperative exercise is safe and can improve shoulder function; however, uncertainty remains regarding the optimal type and dosage, timing of delivery, and effectiveness in terms of cost-benefit ratio of the rehabilitation intervention [9-11].

Among the various rehabilitation approaches, telerehabilitation has shown its effectiveness in ensuring continuity in the care of fragile patients, and after BC surgery during the SARS COVID-19 pandemic period [12]. However, telerehabilitation seems to represent an alternative for all those patients who have difficulty reaching treatment locations, minimizing barriers, such as distance, time, and costs, and facilitating rapid and reducing costs, even after the pandemic era [13,14].

Recent systematic reviews (SRs) have investigated the use of telerehabilitation [15] or eHealth systems [16], such as videoconferencing or counseling and their impact on patients: in the review with meta-analysis conducted by Peng et al [15], telerehabilitation seems to have a significantly positive effect on physical activity (PA), performance, fatigue, and QoL compared with the usual care. Therefore, it is important to highlight that anthropometric body composition and pain did not differ significantly between the 2 groups: these data could confirm the noninferiority of telerehabilitation compared with usual care.

Therefore, eHealth seems to also have a positive impact on psychosocial domains: Wen et al [16] highlight that patients undergoing eHealth have a significantly higher QoL and an improvement in physical and social roles and emotional domains of QoL, even if these subitems have not reached statistical significance.

However, SRs [15-18] have not analyzed the effectiveness of this treatment in improving pain, QoL, or function in this population, despite several studies having been published on the topic [12,19,20]. Therefore, summarizing the results obtained from the studies becomes fundamental to verify the possibility of using this approach as a rehabilitation method for patients post BC surgery.

The main goal of this SR is to investigate the effects of telerehabilitation treatment in women undergoing surgery for BC, in the recovery of QoL. The primary aim is to determine whether telerehabilitation improves QoL compared with (1) conventional, center-based rehabilitation and (2) no rehabilitation. Secondary aims are to assess the effects of telerehabilitation on pain, upper-limb function, shoulder ROM, body composition or weight, and postoperative complication rate, relative to (1) conventional rehabilitation and (2) no rehabilitation.

The hypothesis is that telerehabilitation will be noninferior to conventional rehabilitation and superior to no rehabilitation for QoL; for secondary outcomes, effects will be comparable with conventional care and greater than no rehabilitation, with similar complication rates between telerehabilitation and conventional care, and higher where no rehabilitation is provided.

## Methods

A comprehensive literature review was conducted, accompanied by a meta-analysis, in accordance with the methodological principles set forth in the PRISMA (Preferred Reporting Items for Systematic Reviews and Meta-Analyses) 2020 checklist (Multimedia Appendix 1) [21].

### Search Strategies

A bibliographic search was performed on July 23, 2024, using the following databases: US National Library of Medicine (PubMed or MEDLINE), SCOPUS, Web of Science, and the Cochrane Database of Systematic Reviews. No temporal restrictions were applied for the inclusion of articles. The study question was framed using the Population, Intervention, Comparison, and Outcome (PICO) model [22] available in Table 1. Medical Subject Headings terms and keywords were used for the search string, in accordance with the specifications of each consulted database (Multimedia Appendix 2). To strengthen the research, reference lists from both the selected studies and pertinent literature reviews on the subject were examined as well. The primary goal was to investigate the effects of telerehabilitation treatment compared with other forms of treatment and with no treatment in terms of pain, upper limb functionality, and QoL. The secondary goal was to determine if there is a difference in the rate of complications among those who underwent telerehabilitation treatment, other forms of treatment, or no treatment.

**Table 1:** Population, Intervention, Comparison, and Outcome model.

Domain	Description
Population	Women undergoing surgery for breast cancer
Intervention	Tele-rehabilitation treatment
Comparison	Other treatments, no treatment
Outcomes	Improvement in terms of QoL^a^, pain, function, complications incidence, and body weight composition

^a^QoL: quality of life.

### Eligibility Criteria

This SR included (1) articles in English and Italian; (2) randomized controlled trials (RCTs); (3) studies that included a population of adult women (age >18 y) who underwent surgery for BC; (4) telerehabilitation treatment versus other types of treatment or no treatment; and (5) studies that measured the effects of the treatments in the domains of QoL, pain, function, complications incidence, and body weight composition.

All RCT studies that included a group undergoing telerehabilitation treatment compared with one or more groups undergoing other types of treatment or no treatment were included. Telerehabilitation treatment refers to all rehabilitation interventions delivered remotely, even without direct supervision by health care personnel. Telemonitoring interventions (eg, monitoring of symptom variations such as pain and exercise-related fatigue) were also included.

All publications that had one or more of these elements were excluded: (1) articles written in languages other than English or Italian; (2) non-RCT studies, observational studies, case reports, case series, narrative reviews and overviews, study protocols, SRs, meta-analyses, and gray literature; (3) pediatric population (age <18 y); and (4) conference papers.

### Selection Process

Duplicates were initially identified and removed using the Rayyan web app for SRs [23]. Two reviewers (FS and BC) independently assessed the titles and abstracts to determine their eligibility. At the end of this phase, the 2 reviewers examined, always independently, the full text of the articles to verify their eligibility criteria. After selecting the studies to include, data were extracted. Any discrepancies or disagreements between the 2 reviewers in the previous phases were discussed and resolved with the help of a third reviewer (MB).

### Study Risk of Bias Assessment

The Cochrane risk-of-bias tool for randomized trials version 2 (RoB 2) [24] was used to assess articles included in this SR. To consider all potential areas of bias, the 5 items of the tool (the randomization process, deviations from the intended interventions, missing outcome data, measurement of the outcome, and selection of the reported result) were evaluated as “low,” “some concerns,” or “high.” A proposed judgment about the risk of bias arising from each domain is generated by an algorithm based on answers to the signaling questions. Two authors (FS and BC) independently evaluated the RCTs included. Any disagreements that arose between the primary 2 reviewers were resolved through discussion or with a third reviewer (MB).

### Statistical Analysis

For continuous variables, the mean was calculated according to the guidelines of the Cochrane Handbook for Systematic Reviews of Interventions version 6.3 [25]. If the results were reported as median, IQR, mean value, or 95% CI, a conversion was applied. For studies with incomplete or not directly accessible data, an attempt was made to contact the corresponding author for feedback. In case of no response or inability to provide further data, the articles were excluded from the review. A random-effects model was used a priori due to anticipated clinical and methodological heterogeneity among included studies, including differences in intervention duration and outcome measures. This choice is consistent with recommendations that the use of random-effects models should be based not only on statistical heterogeneity, but also on conceptual considerations [26]. We prespecified subgroup analyses for follow-up time (end of treatment, approximately 12 weeks and 24 weeks) in outcomes reported at multiple timepoints. Where sufficient data were available (≥3 studies per subgroup), we also planned subgroups by outcome instrument family (eg, Functional Assessment of Cancer Therapy vs European Organisation for Research and Treatment of Cancer [EORTC] for QoL) to examine whether the choice of measurement tool influenced pooled effects. Additional subgroup or sensitivity analyses (eg, by comparator type—standard care vs no rehabilitation and by telerehabilitation delivery mode—synchronous, asynchronous, and hybrid) were considered exploratory and conducted only when ≥2 studies informed each category. All analyses were performed using Review Manager (RevMan) version 5.4 (Cochrane Collaboration, 2020), which was used for meta-analyses, forest plots, and chi-square tests for subgroup differences. GRADEpro (guideline development tool; Evidence Prime) was used to generate evidence profiles and a summary of findings.

### Certainty of Evidence

The certainty of evidence for each outcome was evaluated using the Grading of Recommendations Assessment, Development and Evaluation (GRADE) approach [27]. In total, 5 domains were considered: risk of bias, inconsistency, indirectness, imprecision, and publication bias. Evidence profiles and summary of findings tables were generated with the GRADEpro guideline development tool.

## Results

### Study Selection

A total of 4617 studies were found after the systematic search of online databases. From duplicate analysis conducted using Rayyan online software, 1414 articles were deleted.

After the analysis of title, abstract, and keywords, 3045 articles were excluded since they did not meet the inclusion criteria (background article: 1777, wrong outcome: 527, wrong publication type: 383, wrong study design: 253, wrong population: 133, wrong drug: 43, and foreign language: 11). Therefore, 158 articles were analyzed in detail through full text screening to verify their eligibility: 37 studies were excluded due to the publication type (ie, study protocol), 14 were excluded because the study design did not meet the inclusion criteria, 31 articles were excluded because their population was different from patients undergoing BC surgery, 33 articles were excluded because their intervention did not meet the PICO question, and 32 articles were excluded because the outcomes did not meet the PICO question and the inclusion criteria. At the end of the screening process, 11 RCTs [28-38] were selected as they met all the eligibility criteria.

**Figure 1:**
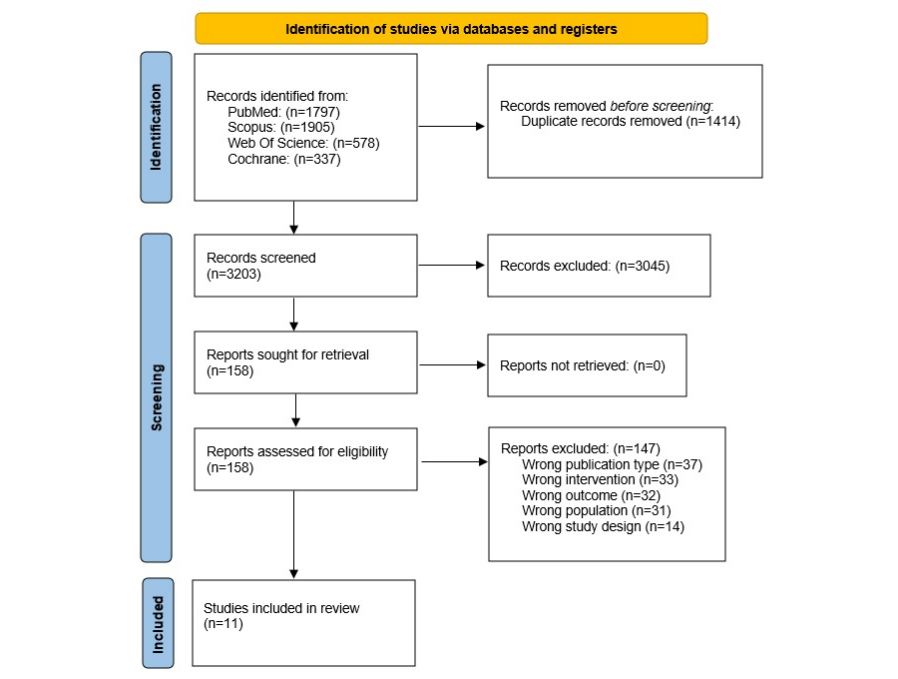
PRISMA (Preferred Reporting Items for Systematic Reviews and Meta-Analyses) flowchart.

The study selection process and the included trials are reported in Figure 1.

### Study Characteristics

A total of 11 RCT studies [28-38] were included and summarized in this SR.

The articles were published between 2016 [30] and 2023 [36,37]. A total of 748 women who underwent BC surgery were enrolled in the control and experimental groups. Table 2 summarizes the sociodemographic and clinical characteristics of participants across the included studies.

**Table 2:** Sociodemographic characteristics.

Study	Country	N		Age (y), mean (SD)		Gender	Cancer stage, n (%)	Surgery type, n (%)
		Intervention	Control	Intervention	Control			
Çınar et al [28]	Turkey	31	33	45.7 (9.0)	45.9 (8.3)	Women only	1: 9 (29) vs 12 (36.4) 2: 12 (38.7) vs 12 (36.4) 3: 10 (32.3) vs 9 (27.2)	Mastectomy 38.7% (12/31) vs 36.4% (12/33) BCSa 61.3% (19/31) vs 63.6% (21/33)
de Aviz et al [38]	Brazil	24	20	48 (6)	52 (7)	Women only	1-3 (eligibility)	Mostly quadrantectomy Axillary dissection frequent
Dong et al [29]	China	26	24	48.0 (5.5)	51.6 (7.5)	Women only	1: 6 (23.1) vs 10 (41.7) 2: 18 (69.2) vs 10 (41.7) 3: 2 (7.7) vs 4 (16.7)	Not specified
Galiano-Castillo et al [30]	Spain	40	41	47 (10)	46 (10)	Women only	1: 28 (34.6) 2: 42 (51.9) 3A: 10 (13.5)	Lumpectomy 42% (34/81) Quadrantectomy 34.6% (28/81) Mastectomy 23.5% (19/81)
Galiano-Castillo et al [31]	Spain	40	41	47 (10)	46 (10)	Women only	1: 28 (34.6) 2: 42 (51.9) 3A: 10 (13.5)	Lumpectomy 42% (34/81) Quadrantectomy 34.6% (28/81) Mastectomy 23.5% (19/81)
Hou et al [32]	Taiwan	53	59	—^b^	—	Women only	2 (most frequent): 44 (39.3)	BCS 67.9% (76/112) Chemo 65.2% (73/112) RTb 66.1% (74/112) Endocrine 53.6% (60/112)
Loubani et al [33]	Israel	18	17	48.0 (11.1)	52.1 (12.8)	Women only	1: 2 (11.1) vs 6 (35.3) 2: 10 (55.6) vs 5 (29.4) 3: 6 (33.3) vs 6 (35.3)	Lumpectomy 55.6% (10/35) vs 76.5% (13/35) Mastectomy 44.4% (8/35) vs 23.5% (4/35)
Ochi et al [34]	Japan	25	25	48 (6)	49 (5)	Women only	1: 36 (72) 2A: 14 (28)	Not specified
Park et al [35]	South Korea	50	50	42.6 (9.1)	47.3 (8.6)	Women only	Not reported	ALNDc 44% (22/100) vs 34% (17/100) Immediate reconstruction 78% (78/100) both groups
Swartz et al [36]	United States	10	10	63.8 (6.4)	63.8 (6.4)	Women only	Stage 0: 2 (11.1) 1: 8 (44.4) 2: 4 (22.2) 3: 4 (22.2)	Mixed, not detailed (with adjuvant CTd/RT)
Zhou et al [37]	China	56	55	49.84 (8.8)	49.98 (9.8)	Women only	1: 34 (30.6) 2: 59 (53.2) 3: 18 (16.2)	MRMe 36% (40/111) Total mastectomy 45.9% (51/111)

^a^BCS: breast-conserving surgery.

^b^Not available.

^b^RT: radiotherapy.

^c^ALND: axillary lymph node dissection.

^d^CT: chemotherapy.

^e^MRM: modified radical mastectomy.

The interventions analyzed were highly heterogeneous regarding the types of instruments used, training frequency, intensity, and duration. However, they can be classified according to the method of therapy delivery.

In total, 5 studies [28,29,32,34,38] based their interventions on mobile apps, albeit in different therapeutic approaches. For instance, Çınar et al [28] designed a mobile app that offered information, advice, and consultations from qualified nurses, alongside daily reminders. In contrast, Dong et al [29] used a step-tracking app for monitoring PA, complemented by remote video instructions for muscle training and supportive messages via social media, while de Aviz et al [38] performed an asynchronous treatment with the sending of exercises via instant messaging app, in order to induce physical improvements, in the QoL and reduce fatigue and pain in postoperative patients with BC.

Only the studies of Galiano-Castillo et al [30,31] focused on a web-based intervention, using the e-CUIDATE system, which provided a personalized exercise program accessible through a secure online interface, while Zhou et al [37] used a multimodal care program via WeChat (Tencent Holdings Limited), including relaxation training, expression of feelings, and social support.

The remaining studies [33,35] combined several rehabilitation interventions: Park et al [35] used the Xbox One Kinect (Microsoft), integrating it with virtual reality experiences; Loubani et al [33] blended clinical sessions with home-based telerehabilitation through the Kinect-based CogniMotion system (ReAbility Online, Gertner Institute, Sheba Medical Center, Ramat Gan, Israel) for both motor and cognitive exercises, while Swartz et al [36] combined virtual exergaming via SecureVideo with PA behavioral coaching.

It is important to highlight that nearly all the studies provided patient education within their interventions. This may include individualized information delivered through online coaching sessions [36], content available via mobile apps [28,29,32] or instant messaging apps [38], or combined methods using email and apps [34], as well as online consultations using platforms such as WeChat [37], exercise guidelines, or written recommendations [35]. Galiano-Castillo [30,31] reported having conveyed the significance of the exercise program in the context of post-BC rehabilitation to the study cohort.

Therefore, the studies proposed a variety of exercises as part of their rehabilitative interventions for women with BC. Among these, the main types include video game–based PA [36], specific exercises for the upper limbs [29,33,35,37,38] or lower limbs, cardiorespiratory training [29], high-intensity interval training [34], relaxation exercises and guided imagination [28], and both resistance and aerobic training [30,31]. Naturally, the choice of exercises depends on the specific objectives of the study, such as improving physical function, QoL, or ROM.

Table 3 provides a comprehensive summary of the RCTs included in this review, detailing study design, sample characteristics, type and duration of interventions, comparators, outcome measures, and main findings.

**Table 3:** Summary table for randomized controlled trials.

Study	Design and aim	Participants			Intervention			Outcome		Results
		Exp^a^	Ctrl^b^	Exp		Ctrl				
Çinar et al [28]	Design: RCTc. Aim: To determine the effect of mobile app–based training on the QoLd in women with breast cancer using endocrine hormonal therapy (EHT).	n=31; mean age 46 (SD 8) y	n=33; mean age 45 (SD 9) y	Routine care plus the access to the educational contents and relaxation exercises and guided imagery applications on the mobile app whenever they wished. The treatment group received supportive care for 12 weeks including symptom management and specialist nurse counseling.		Routine care.	Timepoints: baseline, before intervention (T0), and after 12 weeks (T1). QoL: FACT-ESe Quality of Life Scale; Distress Thermometer Scores		Exp improved QoL after intervention (*P*=.001) while ctrl decreased (*P*=.003). Similarly, distress improved in exp group and between group significant difference was found (*P*=.03).	
de Aviz et al [38]	Design: RCT. Aim: To test the effects of telerehabilitation on upper limb functionality, muscle strength, shoulder range of motion (ROM), QoL, pain and fatigue against an in-person rehabilitation in postoperative patients with breast cancer.	n=24; mean age 48 (SD 6) y	n=20; mean age 52 (SD 7) y	Patients received exercise videos (same exercises as ctrl) via instant messaging apps including active-free shoulder exercises (flexion, extension, abduction, adduction, and rotations), use of ball and wall support (early postoperative 0-15 days). Afterwards, patients received a structured telerehabilitation protocol, also via instant messaging including active-free and resistance exercises for upper and lower limbs, and aerobic exercises. Weekly video calls were implemented to monitor adherence, answer questions, and check for adverse events. Intervention duration: 4 weeks. Frequency: 2 sessions per week (8 sessions total).		Patients received a printed explanatory leaflet with guidelines for postoperative care, including home exercises (same as exp), recommendations for arm care on the surgical side (early postoperative 0-15 days). Afterwards, patients began face-to-face physiotherapy sessions, which included active-free and resistance exercises for upper and lower limbs, aerobic exercises. Intervention duration: 4 weeks. Frequency: 2 sessions per week (8 sessions total).	Timepoints: baseline (T0), and at 4 weeks (end of treatment; T1). QoL: FACT-Gf. Pain: VASg Upper-extremity functioning: DASHh, shoulder muscle strength tested with an isometric dynamometer. Fatigue: FACT-Fi		Between-group comparison showed better results in exp for FACT-G (*P*=.002), pain (*P*=.004), DASH (*P*=.001), FACT-F (*P*=.001), shoulder flexion (*P*=.01), and abduction (*P*=.03). No between group differences for the other muscle strength tested.	
Dong et al [29]	Design: RCT. Aim: To estimate the effects of a combined exercise intervention based on internet and social media software (CEIBISMS) on QoL, muscle strength and cardiorespiratory capacity in postoperative patients with breast cancer.	n=26; mean age 48 (SD 6) y	n=24; mean age 52 (SD 7) y	Muscle training consisting of face-to-face televideo instruction on physical exercise rehabilitation was performed 3 times per week in 30-min sessions, including 5 min warm-up, 20 min muscle training, and 5 min relaxation. Each time; cardiorespiratory training (4 sessions/week, step-recording app); rehabilitation knowledge pushed daily via social media apps. Started after surgery but not specified the exact day of initiation. Intervention duration: 12 weeks.		Traditional treatment and rehabilitation according to daily specifications of the hospital. Started after surgery but not specified the exact day of initiation. Intervention duration: 12 weeks.	Timepoints: baseline (T0) and after 12 weeks (T1). QoL: SF-36j. Functional: SPSDCTk; Muscle strength: ALTl; Cardiorespiratory capacity: VO2maxm.		Exp group improves significantly respect to ctrl in SF-36 subdomain of vitality (*P*=.009), mental health (*P*=.001), and reported health transition (*P*=.048). Similarly, exp group improves significantly in SPSDCT at T1 with respect to ctrl group (*P*=.001) in ALT (*P*=.02). No between group differences for VO2max (*P*=.15).	
Galiano-Castillo et al [30]	Design: RCT. Aim: To compare a telerehabilitation program with usual care in breast cancer survivors.	n=40; mean age 47 (SD 10) y	n=41; mean age 46 (SD 10) y	Telerehabilitation program through the e-CUIDATE system, an online system that facilities the development of remote rehabilitation. The intervention was managed by CUIDATE research staff to assign and check different exercise programs. The schedule consisted of 3 sessions/wk (on nonconsecutive days) that lasted nearly 90 min/d. The intensity and volume of the physical exercise training were established following the recommendations of the American College of Sports Medicine for cancer survivors. Each session contained a battery of specific exercises that were divided into 3 sections: (1) warm-up, (2) resistance and aerobic exercise training, and (3) cooldown. Intervention duration: 8 weeks.		Basic recommendations for exercise in written format. Started after surgery. The exact day of initiation not specified. Intervention duration: 8 weeks.	Timepoints: baseline (T0), after 8-weeks (T1), and after 6 months (T2). QoL: EORTCn QLQ-C30o Pain: Brief pain inventory Upper-extremity functioning: hand grip dynamometer strength test. Fatigue: R-PFSp		Exp showed significantly higher improvements in QoL of the domain of global health status, physical functioning (both *P*=.001), role functioning (*P*=.001), cognitive functioning, and arm symptoms (both *P*=.002) compared with the ctrl at T1. At T2 was observed a maintenance of effects (all *P*=.05) except for role functioning (*P*=.17). At T1, the exp group reported significantly lower pain severity (*P*=.001) and pain interference (*P*=.045) than the ctrl group. At T2, significant results were maintained only for pain interference (*P*=.008). At T1, exp handgrip strength on the affected and nonaffected sides improved significantly (both *P*=.006) compared with the ctrl. This improvement was only maintained at the 6-month follow-up assessment on the affected side (*P*=.03) At T1, exp improved total fatigue perception significantly (*P*=.001) compared with the ctrl. This improvement was at T2 (*P*=.002).	
Galiano-Castillo et al [31]	Design: Secondary analysis of RCT. Aim: To compare a telerehabilitation program with usual care in breast cancer survivors.	n=40; mean age 47 (SD 10) y	n=41; mean age 46 (SD 10) y	Telerehabilitation program through the e-CUIDATE system, an online system that facilities the development of remote rehabilitation. The intervention was managed by CUIDATE research staff to assign and check different exercise programs. The schedule consisted of 3 sessions/wk (on nonconsecutive days) that lasted nearly 90 min/d. The intensity and volume of the physical exercise training were established following the recommendations of the American College of Sports Medicine for cancer survivors. Each session contained a battery of specific exercises that were divided into 3 sections: (1) warm-up, (2) resistance and aerobic exercise training, and (3) cooldown. Intervention duration: 8 weeks.		Basic recommendations for exercise in written format. Started after surgery. The exact day of initiation not specified. Intervention duration: 8 weeks.	Timepoints: baseline (T0), after 8-weeks (T1), and after 6 months (T2). Functional capacity: 6MWTq		Exp group had significantly improved distances (*P*<.001) compared with the ctrl group at T1. At T2, the effect was maintained (*P*=.001).	
Hou et al [32]	Design: RCT. Aim: To evaluate the effectiveness of a breast cancer self-management support (BCSMS) app on the QoL of women with breast cancer.	n=53; mean age 50-64 y	n=59; mean age 50-64 y	BCSMS^r^ mobile app, started after pretest data collection (baseline). Participants could use the app any time as needed. The app was developed with 8 main features, feature number 2 was “exercise and rehabilitation” after surgery. Intervention duration: 3 months.		Received the same care from health care professionals as the experimental group. Intervention duration: 3 months.	Timepoints: baseline (T0), 1.5 months (T1), and 3 months (T2). QoL: EORTC QLQ-C30 and EORTC QLQ-BR23s.		Exp showed significantly higher mean total QoL summary scores from the EORTC QLQ-C30 (83.45 vs 82.23, *P*=.03) and the QLQ-BR23 (65.53 vs 63.13, *P*=.04) at T2 compared with the ctrl. Both groups showed improvements in QoL and symptom scores.	
Loubani et al [33]	Design: RCT. Aim: To examine the effectiveness of a hybrid occupation-based intervention (MaP-BC) on improving daily participation upper limb function and QoL.	n=18; mean age 48 (SD 11.1) y	n=17; mean age 52 (SD 12.8) y	Hybrid managing participation with breast cancer (MaP-BC^t^) consisting of 12 alternating weekly in-clinic and telerehabilitation sessions. Started 3-24 months post-BC^u^ diagnosis. Intervention duration: 6 weeks.		Standard care only. Intervention duration: 6 weeks.	Timepoints: baseline (T1), 6 weeks (T2), and 12 weeks (T3). Participation: COPMv. Activity levels: ACSw. QoL: FACT-Bx. Upper-extremity functioning: DASH; grip strength		Significant interaction (group×time) effects were found for the primary outcome in COPM performance (*P*=.001) and performance satisfaction (*P*=.02). Exp had greater improvement between T1 and T2 in performance and QoL compared with the ctrl group. Both groups improved DASH over time (P<0.001) but with no group×time effect (*P*=.16). Grip strength showed no time (*P*=.17) and no group×time (*P*=.21) effect.	
Ochi et al [34]	Design: RCT. Aim: To determine whether a home-based smartphone-supported high intensity interval training (HIIT) improves cardiorespiratory fitness (CRF) in early- stage breast cancer survivors.	n=25; mean age 48 (SD 6) y	n=25; mean age 49 (SD 5) y	Home-based HIIT^y^ and behavioral modification (Habit-b) with counseling or exercise guidance and support provided via personalized email and a newly developed smartphone app. Intervention duration: 12 weeks (3/week).		Self-monitoring using the wearable device, a smart-watch (Fitbit Versa [Google]).	Timepoints: baseline (T0) and 12 weeks (T1). QoL: EQ-5D total score. Upper-extremity functioning: Grip strength: assessed through a dynamometer. Body composition: BMI; body mass (kg)		No between-group differences in QoL at T1 (*P*=.25). Both groups show improvements in QoL (exp: –0.03; ctrl –0.06). No between-group differences in body composition both for body mass and BMI at T1 (*P*=.25) and for grip strength (*P*=.53).	
Park et al [35]	Design: Multicenter RCT. Aim: To evaluate the effectiveness in improving shoulder ROM, pain, function and QoL of a telerehabilitation therapy using an ARz-based, real-time interactive digital health care system (UINCARE Home+) with a brochure-based home rehabilitation program in postoperative patients with breast cancer.	n=50; mean age 43 (SD 9) y	n=50; mean age 47 (SD 9) y	Telerehabilitation was provided through the AR-based UINCARE Home and rehabilitation system which uses the Xbox One Kinect for Windows. The exercise program consisted of 2 parts with 2 difficulty levels each (total of 4 levels). Each exercise level was composed of warm-up, main workouts, and cool-down components. The exercise level was determined according to the results obtained over the first 4 weeks. Intervention duration 8 weeks.		Brochure detailing the same daily exercise program used for the exp group. The brochure included pictures and brief descriptions of each exercise. The exercise difficulty level was determined according to the results obtained over the first 4 weeks. Intervention duration 8 weeks.	Timepoints: baseline (T0), 4 weeks (T1), 8 weeks (T2), and 12 weeks (T3). QoL: FACT-B; EQ-5D-5L scores Upper-extremity functioning: Shoulder abduction and flexion ROM; QuickDASHaa score Pain: NRSab		All outcomes improved over time. No between-group (group×time) differences for shoulder active and passive ROM: active (*P*=.15) and passive flexion (*P*=.68); and active (*P*=.41) and passive abduction (*P*=.44). Similarly, QuickDASH revealed no between group differences (*P*=.97). No significant group differences were observed for pain (*P*=.56) and for most intense pain (*P*=.64). Similarly, no significant group differences were detected in the EQ-5D-5L or FACT-B scores (*P*=.14 and *P*=.24, respectively)	
Swartz et al [36]	Design: Pilot RCT. Aim: To determine feasibility, acceptability and estimate the effects on physical activity (PA) and physical function of an Active Video Game–Based Physical Activity (AVG-PA) intervention in breast cancer survivors.	n=10; mean age 64.9 (SD 8) y	n=10; mean age 62.6 (SD 4.2) y	12 weekly virtual sessions consisting of AVG-PA^ac^ (Pink Warrior 2), PA^ad^ behavioral coaching, and survivorship navigation discussions.		12 weekly telephone-based breast cancer support group discussions. Participants were provided a Xiaomi Mi Band 3 which delivered progressive step goal notifications and a “reach goal” badge when a person reaches the step goal.	Timepoints: at baseline, week 6, and week 13. Physical activity: average daily steps, average minutes of MVPAae measured by accelerometers. Physical Function: gait speed, SPPBaf, TUGag test, and 2-minute step.		Exp showed moderate effect sizes for (1) SPPB: Cohen d=1.06 and (2) 2-minute step test Cohen d=0.61; and small effect sizes for (1) gait speed Cohen d=0.46, (2) TUG Cohen d=0.43, and (3) MVPA Cohen d= 0.27. ActiGraph data from both groups showed higher MVPA, but lower step counts were found among the exp group participants.	
Zhou et al [37]	Design: RCT. Aim: To investigate the benefits of a WeChat-based multimodal nursing program on health-related quality of life (HRQoL) in postoperative women with breast cancer.	n=56; mean age 49.84 (SD 8.85) y	n=55; mean age 49.98 (SD 9.84) y	WeChat-based multimodal nursing program, including individualized information, surgical side upper limb exercise, psychological guidance, and social support, starting from hospital admission to 6 months post surgery.		Routine nursing care including health education, monitoring, and drainage tube care.	Timepoints: at presurgery, 1-, 3-, and 6-months post surgery QoL: FACT-Bv4.0ah Pain: NRS Fatigue: NRS Sleep: NRS		Exp showed higher improvement than ctrl in total FACT-Bv4.0 scores: *P*<.001 (group), and *P*=.001 (group×time). Pain had only a group-time interaction effect (*P*=.01). No differences for fatigue (*P*=.24) and sleep (*P*=.70).	

^a^Exp: experimental group.

^b^Ctrl: control group.

^c^RCT: randomized controlled trial.

^d^QoL: quality of life.

^e^FACT-ES: Functional Assessment of Cancer Therapy-Endocrine Subscale.

^f^FACT-G: Functional Assessment of Cancer Therapy-General.

^g^VAS: visual analog scale.

^h^DASH: Disability of Arm Shoulder and Hand Questionnaire.

^i^FACT-F: Functional Assessment of Cancer Therapy-Fatigue.

^j^SF-36: Short Form Health Survey 36.

^k^SPSDCT: stand-up and sit-down chair test.

^l^ALT: arm lifting test.

^m^VO2max: maximal oxygen consumption.

^n^EORTC: European Organisation for Research and Treatment of Cancer.

^o^QLQ-C30: Quality-of-Life Questionnaire Core 30.

^p^R-PFS: Piper Fatigue Scale revised.

^q^6MWT: 6-Minute Walk Test.

^r^BCSMS: breast cancer self-management support.

^s^QLQ-BR23: Breast Cancer–Specific Quality-of-Life Questionnaire.

^t^MaP-BC: hybrid managing participation with breast cancer.

^u^BC: breast cancer.

^v^COPM: Canadian Occupational Performance Measure.

^w^ACS: Activity Card Sort.

^x^FACT-B: Functional Assessment of Cancer Therapy-Breast.

^y^HIIT: high intensity interval training.

^z^AR: augmented reality.

^aa^QuickDASH: Quick Version of Disability of Arm Shoulder and Hand Questionnaire.

^ab^NRS: numeric rating scale.

^ac^AVG-PA: active video game–based physical activity.

^ad^PA: physical activity.

^ae^MVPA: moderate to vigorous physical activity.

^af^SPPB: Short Physical Performance Battery.

^ag^TUG: Timed Up and Go.

^ah^FACT-Bv4.0: Functional Assessment of Cancer Therapy-Breast version 4.0.

The characteristics of the included interventions were further synthesized according to the Frequency, Intensity, Time, and Type framework, as reported in Table 4. While frequency and time or duration were consistently described across studies, intensity was often poorly defined or heterogeneous. Notably, in the study by Park et al [35], intensity was not expressed through quantitative measures (eg, load, percentage of one-repetition maximum, or rating of perceived exertion [RPE]) but only described in terms of progression in ROM and the introduction of active movements with or without dumbbells.

**Table 4:** Frequency, intensity, time, and type summary of telerehabilitation interventions across the included randomized controlled trials. The table reports the delivery mode or technology, exercise type, frequency, intensity, duration, supervision or adherence strategies, and comparator for each study.

Study	Delivery mode or technology	Type	Frequency	Intensity	Time or duration	Supervision or adherence	Comparator
Çınar et al [28]	mHealth^a^ app and nurse support	Education, lifestyle, relaxation	On-demand	—^b^	12 weeks	Nurse support; app logs	Routine care
de Aviz et al [38]	Instant message app (exercise video or exercise protocol)	ROM^c^ upper and lower limbs, resistance, and aerobic training	2 times per week	Moderate (Borg 0-10)	Approximately 40 min/session; 8 weeks	Weekly video call	Printed manual + in-person rehabilitation
Dong et al [29]	Face-to-face televideo and social media app	Education, strengthening, endurance, muscle function, and cardiorespiratory training	Daily	RPE^d^ to assess the cardiorespiratory training	30-40 min/day; 12 weeks	Daily remote check	Traditional treatment and rehabilitation
Galiano-Castillo et al [30]	e-CUIDATE system (online system)	Aerobic, resistance, and flexibility	3 times per week	Following recommendations of the ACSM^e^ for cancer survivors	Approximately 90 min/session; 8 weeks	Live supervision and adherence	Printed information on stress management and physical fitness
Galiano-Castillo et al [31]	e-CUIDATE system (online system)	Aerobic, resistance, and flexibility	3 times per week	Following recommendations of the ACSM for cancer survivors	Approximately 90 min/session; 8 weeks	Live supervision and adherence	Printed information on stress management and physical fitness
Hou et al [32]	Mobile app and weekly video calls	ROM, stretching, and strengthening	5 times per week	Low	Approximately 30 min/session; 12 weeks	Weekly check	Printed exercises
Loubani et al [33]	Videoconference (CogniMotion tele-system) and in-clinic session	ROM, stretching, resistance, cognitive abilities	Daily + 2 weekly check	Moderate	Approximately 45 min/session; 6 weeks	Live supervision	Standard care
Ochi et al [34]	Habit-B app, tracker, and email	HIIT^f^ training and education	Daily + weekly check	High	20-30 min/day; 12 weeks	App and wearable logs	Self-monitoring using the wearable device
Park et al [35]	UINCARE Home and rehabilitation system (app and motion sensor)	ROM, strengthening, posture	5 times per week	No information	Approximately 30-40 min/session; 8 weeks	Wearable logs and feedback	Leaflet
Swartz et al [36]	Zoom (Zoom Communications) live sessions and HR^g^ tracker	behavioral coaching, exergames-based activity ( “Mind-body” “e-fitness–based”), and BC^h^ support	3 times per week	Moderate to high	Approximately 45-60 min/session; 12 weeks	Live supervision	Telephone-based support group discussions
Zhou et al [37]	WeChat and routine nursing care	ROM, stretching, strengthening, education	Daily	Low to moderate	6 months	Weekly calls; app logs	Routine nursing care

^a^mHealth: mobile health.

^b^Not applicable.

^c^ROM: range of motion.

^d^RPE: rating of perceived exertion.

^e^ACSM: American College of Sport Medicine.

^f^HIIT: high intensity interval training.

^g^HR: heart rate.

^h^BC: breast cancer.

### Sociodemographic Characteristics

Across the 11 RCTs, a total of 748 women were included (373 allocated to telerehabilitation and 375 to control arms). The mean age of participants spanned from the early 40s to the mid-60s, with most cohorts representing middle-aged women, although the US trial (Swartz et al [36]) recruited an older sample (mean 63.8 y). Cancer stage was predominantly early disease (stages 1-2), although several trials [36] also included patients at stage 3 or 3A. Surgical management varied: breast-conserving surgery predominated in Taiwan (76/112, 67.9%) [32], whereas Turkish and Israeli cohorts [28,33] reported mixed lumpectomy and mastectomy distributions, and immediate reconstruction was frequent in the Korean multicenter trial (78/100, 78%) [35]. Axillary lymph node dissection was reported in the intervention group in the study by Park et al [35]. The 2 Spanish trials [30,31] recruited BC survivors who had completed adjuvant therapy, with a balanced distribution of lumpectomy, quadrantectomy, and mastectomy. In other cohorts, details of surgical procedures were often sparse. Beyond age, sex, stage, and surgery, additional sociodemographic descriptors (eg, education, employment, marital status, ethnicity) were underreported. Where available, most participants were married (69.1%), had a basic educational level (45.7%), and were housewives or on medical leave [31]. Overall, baseline characteristics were generally well balanced between intervention and control groups within studies, supporting internal validity. A full study-level summary is provided in Table 2.

### QoL

In total, 9 articles [28-30,32-35,37,38] assessed QoL as part of their research. The most frequently used assessment was the Functional Assessment of Cancer Therapy-Breast (FACT-B). However, the specific version of the scale varied across studies. For instance, Zhou et al [37] used version 4 of the FACT-B scale, while Loubani et al [33] and Park et al [35] used the original version. Meanwhile, Çınar et al [28] opted for a different approach, using the Functional Assessment of Cancer Therapy-Endocrine Subscale QoL scale, which is tailored for endocrine symptoms and de Aviz et al [38] administered Functional Assessment of Cancer Therapy-General (FACT-G).

The EORTC QoL questionnaires were used in 2 articles [30,32]. Galiano-Castillo et al [30] administered the EORTC Quality-of-Life Questionnaire Core 30 (EORTC QLQ-C30). Similarly, Hou et al [32] also used the EORTC QLQ-C30, but supplemented it with the EORTC Breast Cancer–Specific Quality-Of-Life Questionnaire, a module specifically designed for patients with BC.

The EQ-5D was another instrument used in 2 studies. Ochi et al [34] assessed participants using the standard EQ-5D total score, while Park et al [35] opted for the EQ-5D-5L version.

Only 1 study, conducted by Dong et al [29], used the Short Form Health Survey 36 to evaluate QoL.

As illustrated in Table 3, the majority of studies [28-30,32,33,37,38] reported significant improvements in QoL among participants in the intervention groups. However, not all findings were consistent. Of the total, 2 studies [34,35] did not observe statistically significant differences between the intervention and control groups, even though both groups experienced some degree of improvement.

### Functional and PA

All articles analyzed function and PA, albeit using different evaluation and assessment methods. In general, PA was measured using accelerometers, assessing mean daily steps and mean minutes of moderate to vigorous PA only by Swartz et al [36].

Additionally, Swartz et al [36] used various performance tests to evaluate the effect of their intervention on physical function, including the Short Physical Performance Battery, gait speed, Timed Up and Go test, and 2-minute step test.

Furthermore, 2 other articles used performance tests to measure PA, in particular, Dong et al [29] used the Stand-Up and Sit-Down Chair Test, while Galiano-Castillo et al [31] used the 6-Minute Walk Test (6MWT).

In addition, 3 studies [33,35,38] used questionnaires to assess upper limb function. Specifically, Loubani et al [33] and de Aviz et al [38] used the Disability of Arm, Shoulder, Hand (DASH) questionnaire, while Park et al [35] administered the Quick Version of the Disability of Arm, Shoulder, Hand (QuickDASH) questionnaire score.

Despite the methodologies, results indicated a moderate effect size for the Short Physical Performance Battery and 2-minute step test, along with small effect sizes for gait speed, Timed Up and Go test, and moderate to vigorous PA. Significant improvements were noted in the experimental groups as reported by Dong et al [29], Loubani et al [33], and Galiano-Castillo et al [31].

In contrast, Park et al [35] documented improvements over time without significant differences between the 2 groups.

In total, 5 articles analyzed muscle strength. Specifically, Dong et al [29] evaluated the upper limb strength using the arm lifting test, while De Aviz et al [38] used a digital dynamometer to measure shoulder joint flexion, extension, abduction, external rotation, and internal rotation. Additionally, Loubani et al [33], Ochi et al [34], and Galiano-Castillo et al [30] assessed handgrip strength using a dynamometer.

Strength assessment results indicated significant improvements in the experimental group as reported by Dong et al [29], while de Aviz et al [38] found enhancements in both groups pertaining to upper limb strength, without a statistical difference. Regarding handgrip strength, Galiano-Castillo et al [30] reported significant enhancement solely in the experimental group, which was maintained at the 6-month follow-up. Ochi et al [34] showed no significant differences in between-group analyses.

Of the total, 2 studies [29,31] examined the cardiorespiratory capability: Dong et al [29] used the maximal oxygen consumption, while Galiano-Castillo et al [31] used the 6-Minute Walk Test to evaluate the functional capability. The results were inconsistent; the study by Galiano-Castillo et al [31] showed significant improvements in the intervention group during both T1 and T2 evaluations compared with the control group, while Dong et al [29] found no differences between the groups at the end of the intervention.

ROM was assessed by Park et al [35], specifically focusing on shoulder abduction and flexion and by de Aviz et el [38] for shoulder joint flexion, extension, abduction, external rotation, and internal rotation.

Only Ochi et al [34] evaluated BMI and body mass in kilograms. Both studies reported no significant differences in between-group analyses.

Table 3 presents the results of functional and physical assessments.

### Pain, Fatigue, and Kinesiophobia

In total, 4 articles [30,35,37,38] evaluated pain as an outcome measure. Zhou et al [37] and Park et al [35] used the numerical rating scale (NRS), while de Aviz et al [38] used the visual analog scale, and Galiano-Castillo et al [30] applied the brief pain inventory.

The findings across these studies were variable: 3 studies [30,37,38] reported significantly lower pain in the experimental group than the control group. It is important to underline that, although the results did not present a statistically significant difference between the groups, in the study by de Aviz et al [38], the patients in the experimental group did not present pain at the intermediate and final evaluation.

In contrast, Park et al [35] found no significant differences between the groups.

Fatigue was assessed by Zhou et al [37] using the NRS, Galiano-Castillo et al [30] using the revised Piper Fatigue Scale, and de Aviz et al [38] using the Functional Assessment of Cancer Therapy-Fatigue. Zhou et al [37] reported no significant differences between the groups, while Galiano-Castillo et al [30] and de Aviz et al [38] demonstrated a significant improvement in total fatigue perception in the intervention group compared with the control group, with this effect maintained at follow-up (T2).

Table 3 presents the results of the assessments for pain, fatigue, and kinesiophobia.

### Risk of Bias in Studies

**Figure 2:**
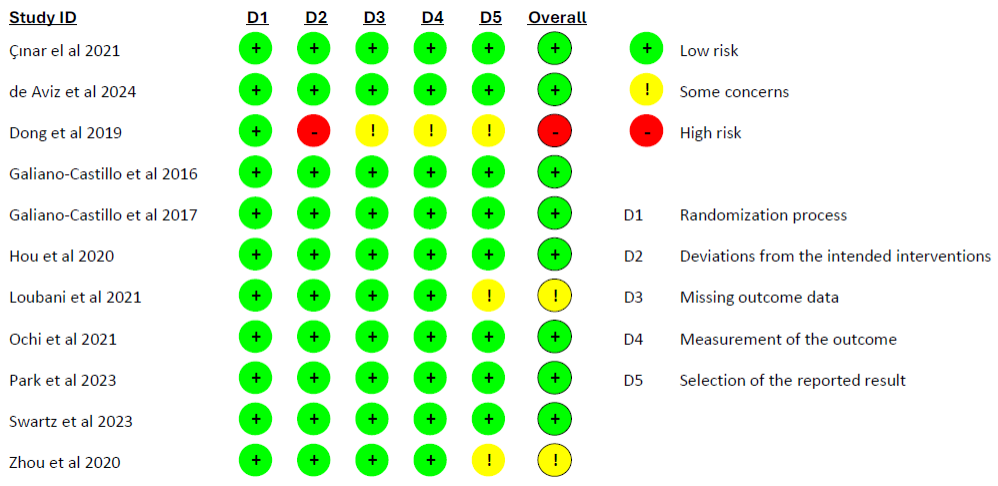
Risk of Bias with Cochrane risk-of-bias tool for randomized trials version 2 [28-38].

The risk of bias assessment with RoB2 [24] across the included studies revealed variability in methodological quality (Figure 2). Most studies, in the overall assessment, are at low risk of bias, while 1 study [33] raises some concerns, and 1 study [29] is at high risk of bias.

Most studies demonstrated a low risk of bias in terms of randomization and allocation concealment, suggesting robust methodological designs. However, 1 study [29] seems to have adopted a per-protocol analysis without a clear balancing of dropouts, raising doubts regarding domain D2, deviations from intended intervention, thus resulting in a high risk of bias. A major limitation across multiple studies was the lack of blinding for both participants and intervention providers, which is particularly relevant given the reliance on patient-reported outcomes in telerehabilitation interventions. This raises concerns about performance and detection bias, potentially influencing the reported effectiveness of interventions. Incomplete outcome data and selective reporting were generally well-managed, with most studies showing a low risk of bias in these domains, ensuring transparency and data integrity. Nonetheless, other biases, such as small sample sizes [36], lack of preregistered protocols in English [33,37], and possible confounding factors, were present in some studies, warranting caution in interpreting their results. Overall, while the included studies provide valuable insights into telerehabilitation for patients post BC surgery, future research should prioritize improved blinding strategies, objective outcome measures, and standardized reporting protocols to enhance methodological rigor and reliability of findings.

### Results of Synthesis

#### Overview

In this meta-analysis, we included only studies [30,33,35,37,38] that evaluated the effectiveness of upper limb muscle strength training via telerehabilitation compared with standard care in patients undergoing surgery for BC. The rigorous selection of articles ensured that only interventions using homogeneous treatment modalities (upper limb muscle strength training) were analyzed, thereby enhancing methodological consistency across the included studies. Regarding standard care groups (control groups), results highlight a significant effect of telerehabilitation based on upper limb muscle strength training on QoL, pain, function, and handgrip strength, demonstrating a clinically relevant improvement compared with standard care at the end of treatment. As for the outcomes related to QoL and pain only, it was possible to analyze long-term effects through follow-up data.

#### QoL

QoL was assessed in 4 studies [30,33,35,37,38] using the FACT-B [33,35,37], FACT-G [38], and EORTC QLQ-C30 [30] at different follow-up points (end of treatment, 12 weeks, and 24 weeks). One additional study [29] was excluded from the meta-analysis due to the use of a noncomparable outcome measure (Short Form Health Survey 36).

**Figure 3:**
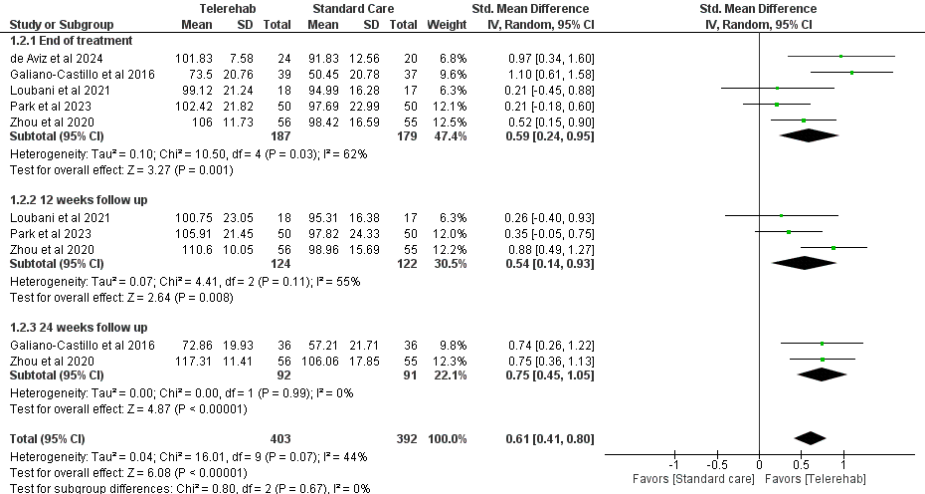
Forest plot of standardized mean differences for the outcome “quality of life” between telerehabilitation and standard care. Results are stratified by time of follow-up assessment. A random-effects model was applied [30,33,35,37,38].

The overall effect across all time points showed a statistically significant improvement in favor of telerehabilitation compared with standard care (standardized mean difference [SMD] 0.61; 95% CI 0.41 to 0.80; *P*<.001), indicating a moderate effect size. Subgroup analysis based on follow-up time (end of treatment, 12 weeks, and 24 weeks) revealed no statistically significant differences between subgroups (χ^2^_2_=0.8, *P*=.67). Each time-point analysis showed statistically significant results in favor of telerehabilitation (Figure 3).

#### Pain

**Figure 4:**
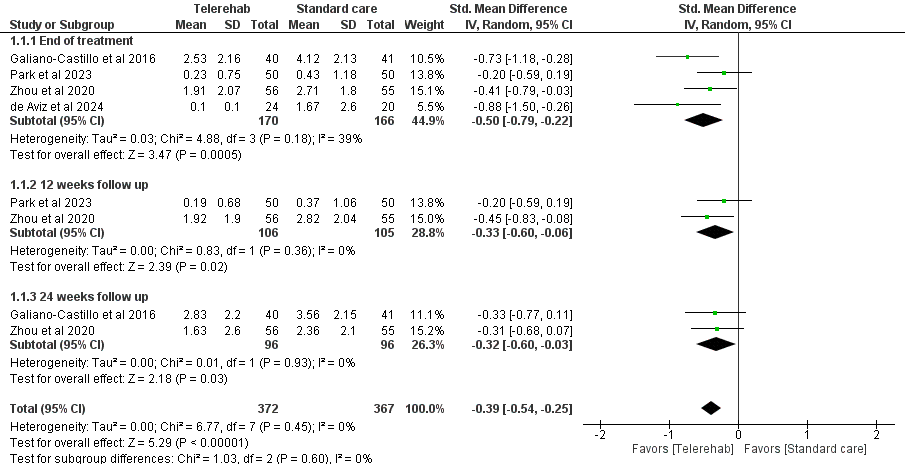
Forest plot of the standardized mean differences for the outcome “pain intensity” between telerehabilitation and standard care. Results are grouped by time of follow-up assessment. A random-effects model was used [30,35,37,38].

In total, 4 studies (n=731) [30,35,37,38] assessed pain using validated measures such as the visual analog scale [38], brief pain inventory [30], and NRS [35,37] at various time points (end of treatment, 12 weeks, and 24 weeks). The overall analysis showed a statistically significant reduction in pain intensity in favor of telerehabilitation compared with standard care (SMD –0.39; 95% CI –0.54 to –0.25; *P*<.001), indicating a small to moderate effect size. Subgroup analysis by follow-up time showed consistent results, with no statistically significant differences between subgroups (*χ*^2^_2_=1.0; *P*=.60). Each subgroup showed a statistically significant effect of telerehabilitation (Figure 4).

#### Upper Limb Function

**Figure 5:**
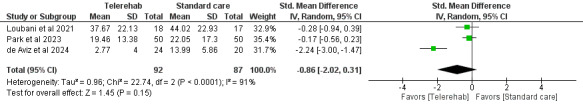
Forest plot of standardized mean difference for the outcome “upper limb function” between telerehabilitation and standard care at the end of the treatment. A random-effects model was applied [33,35,38].

Upper limb function was evaluated in 3 studies (n=171) [33,35,38] at the end of the intervention using DASH [35,38] and QuickDASH scores [33]. The meta-analysis showed no statistically significant difference between telerehabilitation and standard care (SMD –0.86; 95% CI –2.02 to 0.31; *P*=.15), indicating a small and nonsignificant effect in favor of telerehabilitation (Figure 5).

#### Handgrip Strength

**Figure 6:**
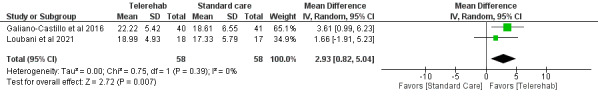
Forest plot of standardized mean difference for the outcome “handgrip strength” between telerehabilitation and standard care. A random-effects model was used [30,33].

In total, 2 studies (n=116) [30,33] examined the effect of telerehabilitation on handgrip strength. Handgrip strength was assessed through a handheld dynamometer at the end of treatment. The overall effect was statistically significant in favor of telerehabilitation (mean difference [MD] 2.93; 95% CI 0.82-5.04; *P*=.006), indicating a moderate improvement (Figure 6).

### Certainty of Evidence

The GRADE summary of findings (Table 5) for the main outcomes at different time points showed that at the end of treatment moderate evidence suggests that telerehabilitation was associated with a statistically significant improvement in QoL compared with standard care (SMD 0.59, 95% CI 0.24-0.95; moderate certainty); similarly regarding to handgrip strength (MD 2.93 kg, 95% CI 0.82-5.04; low certainty). A moderate reduction in pain was also observed (SMD 0.50 lower, 95% CI 0.79-0.22 lower; low certainty). No relevant effects were found for upper limb function (SMD 0.86 lower, 95% CI –2.02 to 0.31; low certainty). During the 12-week follow-up, telerehabilitation maintained a beneficial effect on QoL (SMD 0.54 higher, 95% CI 0.14-0.93; low certainty) and was associated with a reduction in pain intensity (SMD 0.33 lower, 95% CI –0.60 to –0.06; low certainty). At 24 weeks follow-up, the improvement in QoL was further increased (SMD 0.75 higher, 95% CI 0.45-1.05; low certainty), while pain reduction remained modest (SMD 0.32 lower, 95% CI –0.60 to –0.03; low certainty). The certainty of evidence ranged from low to moderate, mainly due to limitations in study design and imprecision related to small sample sizes and wide CIs.

**Table 5:** Grading of Recommendations Assessment, Development and Evaluation summary of findings.

Outcomes		Assessment tool	Number of participants, n (studies)	Certainty of the evidence (GRADE^a^)	Anticipated absolute effects (95% CI), mean value with standard care	MD^b^ or SMD^c^ with telerehabilitation (95% CI)
**End of treatment**						
	QoL^d^	FACT-B^e^; FACT-B V4.0^f^; EORTC^g^ QLQ-C30^h^; FACT-G^i^	366 (5 RCTs^j^)	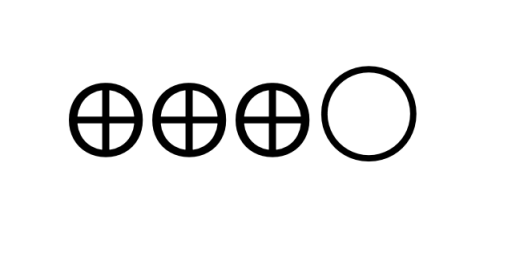 Moderate^k,l^	NE^m^	SMD 0.59 (0.24 to 0.95)
	Pain	VAS^n^; NPRS^o^; BPI^p^	336 (4 RCTs)	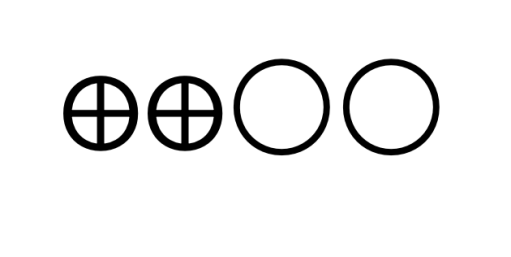 Low^l,q^	NE	SMD –0.50 (–0.79 to –0.22)
	Upper limb function	DASH^r^; quickDASH^s^	179 (3 RCTs)	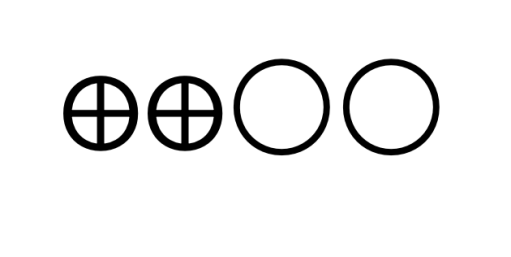 Low^k,l^	NE	SMD –0.86 (–2.02 to 0.31)
	Handgrip strength	Handheld dynamometer	116 (58 RCTs)	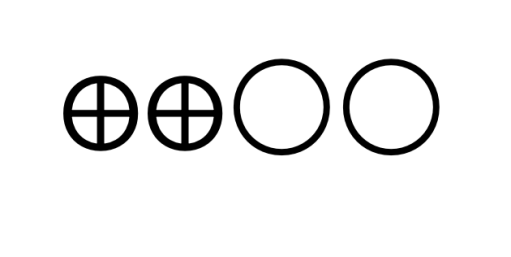 Low^k,l^	The mean handgrip strength was **17.97** kg	MD 2.93 (0.82 to 5.04)
**12 weeks follow-up**						
	QoL	FACT-B; FACT-B V4.0; EORTC QLQ-C30	246 (3 RCTs)	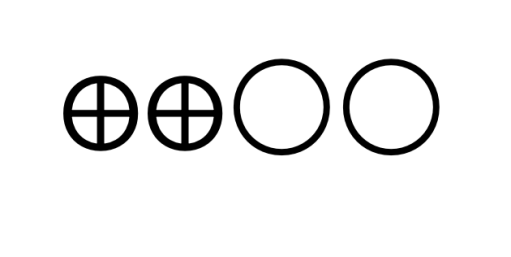 Low^k,l^	NE	SMD 0.54 (0.14 to 0.93)
	Pain	NPRS	211 (2 RCTs)	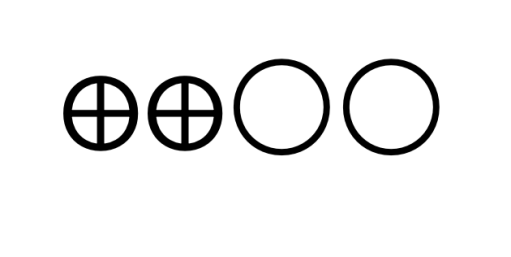 Low^k,l^	NE	SMD –0.33 (–0.6 to –0.06)
**24 weeks follow-up**						
	QoL	FACT-B V4.0; EORTC QLQ-C30	183 (2 RCTs)	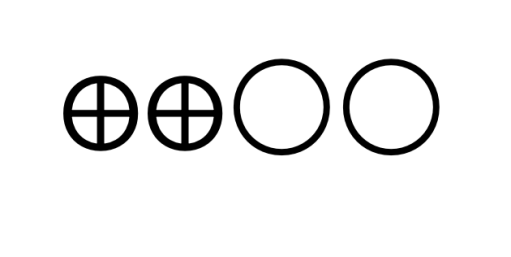 Low^k,l^	NE	SMD 0.75 (0.45 to 1.05)
	Pain	NPRS; BPI	192 (2 RCTs)	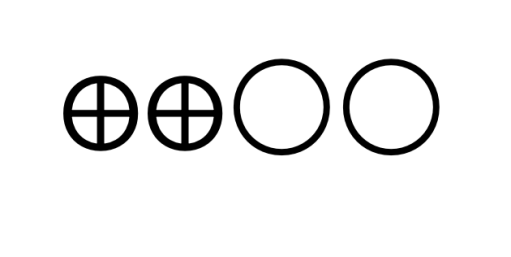 Low^k,l^	NE	SMD –0.32 (–0.6 to –0.03)

^a^GRADE: Grading of Recommendations Assessment, Development and Evaluation. GRADE Working Group grades of evidence. High certainty: we are very confident that the true effect lies close to that of the estimate of the effect. Moderate certainty: we are moderately confident in the effect estimate: the true effect is likely to be close to the estimate of the effect, but there is a possibility that it is substantially different. Low certainty: our confidence in the effect estimate is limited: the true effect may be substantially different from the estimate of the effect. Very low certainty: we have very little confidence in the effect estimate: the true effect is likely to be substantially different from the estimate of effect.

^b^MD: mean difference.

^c^SMD: standardized mean difference.

^d^QoL: quality of life.

^e^FACT-B: Functional Assessment of Cancer Therapy-Breast.

^f^FACT-B V4.0: Functional Assessment of Cancer Therapy-Breast version 4.0.

^g^EORTC: European Organisation for Research and Treatment of Cancer.

^h^QLQ-C30: Quality-of-Life Questionnaire Core 30.

^i^FACT-G: Functional Assessment of Cancer Therapy-General.

^j^RCT: randomized controlled trial.

^k^Lack of details on randomization, allocation concealment, and masking in 1 or + studies.

^l^Total sample size < 400.

^m^NE: not estimable.

^n^VAS: visual analog scale.

^o^NPRS: numeric pain rating scale.

^p^BPI: brief pain inventory.

^q^Lack details on reported results in 1 or + studies.

^r^DASH: Disability of Arm Shoulder and Hand Questionnaire.

^s^quickDASH: Quick Version of Disability of Arm Shoulder and Hand Questionnaire.

## Discussion

### Principal Results

This SR with meta-analysis evaluated the clinical effectiveness of telerehabilitation in women undergoing BC surgery, focusing on 4 core outcomes: QoL, pain, upper limb function, and handgrip strength. A total of 11 RCTs were included in the qualitative synthesis, of which 5 were eligible for meta-analysis. The overall findings support the use of telerehabilitation as a valid, safe, and in some cases superior alternative to routine care, especially in improving QoL and muscular strength, while some functional outcomes require further investigation.

The most consistent benefit of telerehabilitation across included studies was its effect on QoL. The meta-analysis showed a moderate, statistically significant improvement (SMD 0.59) with moderate certainty of evidence. This effect was maintained across multiple follow-up points and QoL instruments, including FACT-B, FACT-G, and EORTC QLQ-C30. These findings align with those reported by Wen et al [16], who observed that eHealth-based interventions significantly improved general and subdomain QoL metrics in women with BC. Similarly, Peng et al [15] noted improvements in fatigue and functional domains in patients with BC who engaged in structured tele-exercise programs.

Specific interventions, such as the e-CUIDATE system studied by Galiano-Castillo et al [30], showed both QoL gains and durability of effects over a 6-month follow-up, supporting the long-term potential of well-structured telerehabilitation protocols.

In contrast, Scaturro et al [12] found that synchronous telerehabilitation, delivered through a web-meeting platform, was effective, but face-to-face rehabilitation yielded greater QoL improvements, potentially due to individualized adjustment and closer supervision. This observation underscores the role of patient-physiotherapist interaction and feedback loops, which are harder to replicate in remote formats.

Pain control emerged as another area where telerehabilitation was effective. The pooled analysis showed a significant reduction in pain (SMD –0.50). In literature, the beneficial effects of rehabilitation and physical exercise on pain reduction in patients undergoing BC surgery are well-established [39]. However, to date, no SRs have comprehensively evaluated this specific outcome in the context of telerehabilitation in this specific population. To the best of our knowledge, this is the first SR with meta-analysis to demonstrate the reduction of pain in patients receiving telerehabilitation following BC surgery.

Generally, the efficacy of telerehabilitation in alleviating pain is documented for both acute [40,41] and chronic pain, short-term and long-term [41], in patients following cancer surgery. The findings of our SR are thus consistent with existing literature on this subject.

Handgrip strength improved significantly (MD 2.93 kg); nevertheless, given the low certainty of evidence and reference values for minimal clinically important differences reported in the literature, this change is unlikely to be clinically meaningful [42,43]. Grip strength is an important functional indicator post mastectomy or quadrantectomy and is correlated with patient independence and upper limb integrity [44].

Upper limb function did not significantly improve in the meta-analysis (SMD –0.86, 95% CI –2.02 to 0.31). This may be due to substantial variability in the tools used (DASH and Quick-DASH), the intensity of training, or baseline impairments. Scaturro et al [12] found outpatient rehabilitation to be more effective than telerehabilitation in improving upper limb functional scores, possibly due to the capacity for individualized correction and targeted kinematic feedback.

Park et al [35] attempted to overcome this by using virtual reality–based telerehabilitation with the Xbox Kinect, achieving encouraging improvements in shoulder function and patient engagement.

The clinical implications of these findings are substantial. Telerehabilitation overcomes several barriers that hamper access to rehabilitation services, including geographic isolation, transportation challenges, financial burdens, and caregiver constraints. Studies such as those by Keikha et al [17] and Anik et al [45] emphasize that telerehabilitation can significantly enhance service reach and continuity of care, particularly for underserved populations.

Furthermore, its cost-effectiveness is becoming increasingly recognized [46-48]. Remote protocols may reduce direct and indirect costs, a hypothesis supported by implementation studies showing decreased health care usage when telerehabilitation is adopted as part of routine cancer recovery [12].

Several trials also incorporated patient education and behavioral coaching components [28,34,36] via messaging apps or interactive video calls (eg, WeChat-based multimodal nursing by Zhou et al [37]), highlighting how multidomain interventions can reinforce adherence and long-term engagement.

The sociodemographic and clinical profiles of participants in the included trials provide important context for interpreting the findings of this SR. Most samples consisted of middle-aged women, with mean ages ranging from the early 40s to the mid-60s. Cancer stage was predominantly early (1-2), although several studies included stage 3 cases, while surgical management varied between breast-conserving surgery, mastectomy, and immediate reconstruction. These characteristics reflect the typical population of BC survivors eligible for rehabilitation, but also highlight potential limitations in generalizability.

A relevant aspect emerging from the FIIT analysis concerns exercise intensity. Unlike frequency and duration, which were usually well defined, intensity was rarely standardized across studies. Some trials referred to perceived exertion scales (eg, Borg and RPE) or to international recommendations (eg, American College of Sport Medicine), while others, such as Park et al [35], described progression exclusively in terms of ROM and the transition from passive to active movements with or without dumbbells, without specifying external load. This heterogeneity, and in some cases the total absence of information, hinders direct comparison between protocols and limits clinical reproducibility. From a methodological perspective, the lack of clear criteria for load progression makes it difficult to determine whether observed improvements were due to training frequency, total volume, or actual modulation of intensity. Future telerehabilitation trials should therefore adopt shared and explicit parameters for defining exercise intensity (eg, percentage of one-repetition maximum, RPE, or progressive load adjustments), in order to ensure greater standardization and clinical transferability.

### Limitations

Despite promising results, this review presents some limitations. Only 5 studies were included in the meta-analysis due to heterogeneity in intervention types, duration, and outcome measures. Sample sizes were often small, and blinding was generally not feasible, increasing the risk of performance and detection bias. Furthermore, most studies lacked long-term follow-up data, making it difficult to assess the sustainability of effects beyond 3-6 months. The GRADE approach revealed moderate certainty for QoL but low certainty for other outcomes, primarily due to methodological limitations and imprecision.

One relevant limitation of this meta-analysis is the heterogeneity among the included telerehabilitation interventions in terms of delivery mode (eg, mobile app, videoconferencing, and SMS text messaging), session frequency, intensity, and duration. Additionally, the standard care comparator varied across studies, encompassing different rehabilitation strategies and intensities. This clinical and methodological variability may have influenced the estimated effect sizes. The use of a random-effects model was a deliberate methodological choice to account for and mitigate such heterogeneity. Future trials should aim to harmonize both intervention and comparator protocols to enhance comparability and validity. Furthermore, the underreporting of sociodemographic variables such as education, employment, or ethnicity in many trials limits our ability to fully explore how social context may interact with the effectiveness of telerehabilitation. Future research should systematically report these descriptors to improve external validity and allow stratified analyses.

### Future Perspectives

From a clinical perspective, these findings support the integration of telerehabilitation into standard postoperative care for women after BC surgery. In particular, telerehabilitation appears to be a valuable complement to in-person rehabilitation, especially for patients who face barriers such as distance, limited mobility, or time constraints. To maximize its effectiveness, clinical programs should be individualized, taking into account disease stage, type of surgery, functional status, and patients’ access to and familiarity with digital technologies. From a research perspective, future trials should prioritize the use of standardized and validated outcome measures to ensure comparability across studies and to strengthen the evidence base for meta-analyses. Furthermore, consistent and detailed reporting of sociodemographic variables is needed, as these factors may significantly influence adherence to telerehabilitation and treatment effectiveness, ultimately affecting the external validity of the findings. Addressing these aspects will enhance both the robustness of future evidence and its applicability in clinical practice. Finally, while this review did not include formal cost-effectiveness analyses, the potential economic implications of telerehabilitation deserve attention. Telerehabilitation may reduce indirect costs related to travel, time, and health care resource usage, making it an attractive option for both patients and health care systems. However, these assumptions require confirmation through well-designed economic evaluations alongside clinical trials. Future studies should therefore integrate cost-effectiveness analyses to determine whether the clinical benefits of telerehabilitation are accompanied by sustainable economic advantages.

### Conclusions

This SR and meta-analysis confirm that telerehabilitation is an effective and viable approach for postoperative care in women undergoing BC surgery. It significantly improves QoL, reduces pain, and enhances grip strength compared with standard care, with moderate to low certainty of evidence. No significant improvement was observed in the upper limb function, although the direction of effect favors telerehabilitation. The variability across interventions and outcome measures suggests a need for more standardized, high-quality trials. Further research should focus on long-term outcomes, technological enhancements, and cost-effectiveness to support broader implementation.

## Appendix

Multimedia Appendix 1 PRISMA 2020 checklist.

Multimedia Appendix 2 Search strategy.

## Abbreviations

BC: breast cancer
DASH: Disability of Arm, Shoulder, and Hand
EORTC: European Organisation for Research and Treatment of Cancer
EORTC QLQ-C30: EORTC Quality-Of-Life Questionnaire Core 30
FACT-B: Functional Assessment Of Cancer Therapy-Breast
FACT-G: Functional Assessment of Cancer Therapy-General
GRADE: Grading of Recommendations Assessment, Development and Evaluation
MD: mean difference
NRS: numeric rating scale
PA: physical activity
PICO: Population, Intervention, Comparison, and Outcome
PRISMA: Preferred Reporting Items for Systematic Reviews and Meta-Analyses
QoL: Quality of life
Quick-DASH: Quick Version of Disability of Arm, Shoulder, and Hand Questionnaire
RCT: randomized controlled trial
RoB2: Cochrane risk-of-bias tool for randomized trials version 2
ROM: range of motion
RPE: rating of perceived exertion
SMD: standardized mean difference
SR: systematic review
