# Supplementary material for: Telerehabilitation in Postoperative Breast Cancer Care: Systematic Review and Meta-Analysis
Source: JMIR Rehabil Assist Technol. 2025 Dec 9;12:e77161. doi: 10.2196/77161 (PMC12728404; doi:10.2196/77161)
Supplement: Multimedia Appendix 2 [file rehab_v12i1e77161_app2.docx]

PubMed:

(“Telerehabilitation”[Mesh] OR “telerehabilitation” OR “Telerehabilitations” OR “Tele-rehabilitation” OR “Tele rehabilitation” OR “Remote Rehabilitation” OR “Rehabilitation, Remote” OR “Virtual Rehabilitation*” OR “Rehabilitation, Virtual” OR “telemonitoring” OR “Telemedicine”[Mesh] OR “telemedicine” OR “Exergaming”[Mesh] OR “exergaming” OR “Virtual Reality Exercise” OR “Active-Video Gaming” OR “exergames” OR “Telecommunications”[Mesh] OR “Telecommunications” OR “telehealth” OR “videoconferenc*” OR “teletreatment*” OR “teletherapy” OR “Distance Education” OR “Telepractice” OR “Virtual Conferenc*”) AND (“Breast Neoplasms”[Mesh] OR “Breast Neoplasm*” OR “Neoplasm, Breast” OR “Breast Tumor*” OR “Tumor, Breast” OR “Tumors, Breast” OR “Neoplasms, Breast” OR “Breast Cancer” OR “Cancer, Breast” OR “Mammary Cancer*” OR “Cancer, Mammary” OR “Cancers, Mammary” OR “Malignant Neoplasm of Breast” OR “Breast Malignant Neoplasm” OR “Breast Malignant Neoplasms” OR “Malignant Tumor of Breast” OR “Breast Malignant Tumor” OR “Breast Malignant Tumors” OR “Cancer of Breast” OR “Cancer of the Breast” OR “Mammary Carcinoma, Human” OR “Human Mammary Carcinomas” OR “Human Mammary Carcinoma” OR “Human Mammary Neoplasms” OR “Breast Carcinoma” OR “Breast Carcinomas” OR “Carcinoma, Breast” OR “Carcinomas, Breast”)

Scopus:

(Telerehabilitation OR Telerehabilitations OR "Tele-rehabilitation" OR "Tele rehabilitation" OR "Remote Rehabilitation" OR "Rehabilitation, Remote" OR "Virtual Rehabilitation*" OR "Rehabilitation, Virtual" OR telemonitoring OR Telemedicine OR telemedicine OR Exergaming OR exergaming OR "Virtual Reality Exercise" OR "Active-Video Gaming" OR exergames OR Telecommunications OR telecommunications OR telehealth OR videoconferenc* OR teletreatment* OR teletherapy OR "Distance Education" OR Telepractice OR "Virtual Conferenc*") AND ("Breast Neoplasms" OR "Breast Neoplasm*" OR "Neoplasm, Breast" OR "Breast Tumor*" OR "Tumor, Breast" OR "Tumors, Breast" OR "Neoplasms, Breast" OR "Breast Cancer" OR "Cancer, Breast" OR "Mammary Cancer*" OR "Cancer, Mammary" OR "Cancers, Mammary" OR "Malignant Neoplasm of Breast" OR "Breast Malignant Neoplasm" OR "Breast Malignant Neoplasms" OR "Malignant Tumor of Breast" OR "Breast Malignant Tumor" OR "Breast Malignant Tumors" OR "Cancer of Breast" OR "Cancer of the Breast" OR "Mammary Carcinoma, Human" OR "Human Mammary Carcinomas" OR "Human Mammary Carcinoma" OR "Human Mammary Neoplasms" OR "Breast Carcinoma" OR "Breast Carcinomas" OR "Carcinoma, Breast" OR "Carcinomas, Breast")

Web of science:

TS=(Telerehabilitation OR telerehabilitation OR Telerehabilitations OR "Tele-rehabilitation" OR "Tele rehabilitation" OR "Remote Rehabilitation" OR "Rehabilitation, Remote" OR "Virtual Rehabilitation*" OR "Rehabilitation, Virtual" OR telemonitoring OR Telemedicine OR telemedicine OR Exergaming OR exergaming OR "Virtual Reality Exercise" OR "Active-Video Gaming" OR exergames OR Telecommunications OR telecommunications OR telehealth OR videoconferenc* OR teletreatment* OR teletherapy OR "Distance Education" OR Telepractice OR "Virtual Conferenc*") AND TS=("Breast Neoplasms" OR "Breast Neoplasm*" OR "Neoplasm, Breast" OR "Breast Tumor*" OR "Tumor, Breast" OR "Tumors, Breast" OR "Neoplasms, Breast" OR "Breast Cancer" OR "Cancer, Breast" OR "Mammary Cancer*" OR "Cancer, Mammary" OR "Cancers, Mammary" OR "Malignant Neoplasm of Breast" OR "Breast Malignant Neoplasm" OR "Breast Malignant Neoplasms" OR "Malignant Tumor of Breast" OR "Breast Malignant Tumor" OR "Breast Malignant Tumors" OR "Cancer of Breast" OR "Cancer of the Breast" OR "Mammary Carcinoma, Human" OR "Human Mammary Carcinomas" OR "Human Mammary Carcinoma" OR "Human Mammary Neoplasms" OR "Breast Carcinoma" OR "Breast Carcinomas" OR "Carcinoma, Breast" OR "Carcinomas, Breast")

Cochrane:

(Telerehabilitation OR telerehabilitation OR Telerehabilitations OR "Tele-rehabilitation" OR "Tele rehabilitation" OR "Remote Rehabilitation" OR "Rehabilitation, Remote" OR "Virtual Rehabilitation*" OR "Rehabilitation, Virtual" OR telemonitoring OR Telemedicine OR telemedicine OR Exergaming OR exergaming OR "Virtual Reality Exercise" OR "Active-Video Gaming" OR exergames OR Telecommunications OR telecommunications OR telehealth OR videoconferenc* OR teletreatment* OR teletherapy OR "Distance Education" OR Telepractice OR "Virtual Conferenc*") AND ("Breast Neoplasms" OR "Breast Neoplasm*" OR "Neoplasm, Breast" OR "Breast Tumor*" OR "Tumor, Breast" OR "Tumors, Breast" OR "Neoplasms, Breast" OR "Breast Cancer" OR "Cancer, Breast" OR "Mammary Cancer*" OR "Cancer, Mammary" OR "Cancers, Mammary" OR "Malignant Neoplasm of Breast" OR "Breast Malignant Neoplasm" OR "Breast Malignant Neoplasms" OR "Malignant Tumor of Breast" OR "Breast Malignant Tumor" OR "Breast Malignant Tumors" OR "Cancer of Breast" OR "Cancer of the Breast" OR "Mammary Carcinoma, Human" OR "Human Mammary Carcinomas" OR "Human Mammary Carcinoma" OR "Human Mammary Neoplasms" OR "Breast Carcinoma" OR "Breast Carcinomas" OR "Carcinoma, Breast" OR "Carcinomas, Breast")
